# A social network analysis on immigrants and refugees access to services in the malaria elimination context

**DOI:** 10.1186/s12936-018-2635-4

**Published:** 2019-01-03

**Authors:** Ensiyeh Jamshidi, Hassan Eftekhar Ardebili, Reza Yousefi-Nooraie, Ahmad Raeisi, Hossein Malekafzali Ardakani, Roya Sadeghi, Ahmad Ali Hanafi-Bojd, Reza Majdzadeh

**Affiliations:** 1 Department of Health Education and Promotion, School of Public Health, Tehran University of Medical Sciences, Tehran, Iran; 2 Community Based Participatory Research Center, Iranian Institute for Reduction of High-Risk Behaviors, Tehran, Iran; 3 Department of Public Health Sciences, University of Rochester, Rochester, USA; 4 Department of Medical Entomology and Vector Control, Tehran University of Medical Sciences, Tehran, Iran; 5 Department of Epidemiology and Biostatistics, School of Public Health, Tehran University of Medical Sciences, Tehran, Iran

**Keywords:** Malaria elimination, Inter-sectoral collaboration, Network analysis, Levels of collaboration, Immigrants, Refugees, Access to service

## Abstract

**Background:**

There has been significant progress in eliminating malaria in Iran. The aim of this study is to investigate the structure of inter-organizational collaboration networks in the field of unauthorized immigrants and refugees access to services in order to eliminate malaria.

**Methods:**

This study employed social network analysis, in which nodes represented stakeholders associated with providing access of immigrants and refugees to services in the field of malaria elimination, and ties indicated the level of collaboration. This study adopted socio-centric analysis and the whole network was studied. In this regard, 12 districts of the malaria-endemic area in Iran were selected. Participants included 360 individuals (30 representatives of the organization/group in each district). The data were gathered by interview, using the levels of collaboration scale. UCINET 6 was used for data analysis. The indices of density, centralization, reciprocity, and clustering were investigated for each twelve network and at each level of collaboration.

**Results:**

The average density of the networks was 0.22 (SD: 0.04). In districts with a high incidence of imported malaria, the values of network density and centralization were high and the networks comprised of a larger connected component (less isolated clusters). There were significant correlations between density of network (r = 0.66, P = 0.02), degree centralization (r = 0.65, P = 0.02), betweenness centralization (r = 0.76, P = 0.004), and imported malaria cases. In general, the degree centrality and betweenness centrality of the organizations of health, district governor, and foreign immigrants’ affairs were higher. In all networks, 60% of the relationships were bilateral. At a higher level of collaboration, the centralization declined and reciprocity increased. The average of betweenness centralization index was 22.76 (SD = 3.88).

**Conclusions:**

Higher values of network indices in border districts and districts with more cases of imported malaria, in terms of density and centralization measures, can propose the hypothesis that higher preparedness against the issue and centralization of power can enable a better top-down outbreak management, which needs further investigations. Higher centrality of governmental organizations indicates the need for involving private, non-governmental organizations and representatives of immigrant and refugee groups. Recognition of the existing network structure can help the authorities increase access to malaria prevention, diagnosis, and treatment services among immigrants and refugees.

**Electronic supplementary material:**

The online version of this article (10.1186/s12936-018-2635-4) contains supplementary material, which is available to authorized users.

## Background

The vision of a world without malaria has been emphasized in the global technical strategy for malaria (2016–2030) and accordingly, at least 35 countries are set to eliminate malaria by 2030 [1]. Nowadays, there are one hundred endemic countries with continuous malaria transmission, in spite of highlighting the concept of eliminating malaria. However, cases of re-introduced disease in malaria-free areas, through population movement, from endemic countries such as the eastern Mediterranean region, as well as spreading drug-resistant parasites, has taken place more than once [2].

Iran is the world’s third country in terms of the number of registered refugees [3]. A roughly estimated 1.5 million illegal immigrants and refugees from endemic neighbouring countries have created a serious threat to the country’s programme developed for eliminating malaria [4, 5].

The need for collaboration has considerably increased in a new era of expanding public health challenges and depleting resources [6]. Networking and partnership building among stakeholders is crucial for adopting, implementing, and sustaining effective community-based programmes [7–9]; and can facilitate integrated efforts for solving public health problems [10–12]. Multiple sectors can share the responsibilities influencing the target population’s health more effectively through communicating and integrating related resources, talents, and strategies [13, 14].

In addition, collaborative efforts and cooperation with different organizations and communities, along borders and in areas with high population mobility, is more effective than merely focusing on the tracking of mobile populations and specific risk groups [15]. In addition, roles of the participating stakeholders become more evident as a disease inches closer to elimination because the public opposition can significantly undermine a disease control programme [16, 17].

Social networks are formed by social interactions between individuals or organizations [18]. Social network analysis (SNA) is a well-established methodology for describing, exploring, and understanding social relationships [11]. It has implications for understanding, guiding, and improving the process of programme implementation [7]. Various indicators of network structure, such as density (the proposition of existing ties), reciprocity (the proportion of mutual ties), and indicators of the proportional prominence of network actors (i.e. centrality measures) provide a snapshot of the social dynamics among the network actors, which can provide a framework to analyse partnership and collaboration among different organizations [19, 20].

Implementing malaria elimination programmes for accessing high-risk populations through social networks and engaging effectively at different points of mobility systems will be more practical, if population mobility is regarded as a system and social process is driven by a range of social, economic, and cultural factors, involving multiple groups [15]. Malaria elimination programme can successfully use social networks to access mobile contractors [21], communicate with unregistered workers, and recruit immigrants to work as community health volunteers [22]. These social networks can establish a point of access to the target population as well as help the researchers identify other potential sites of transmission, by which they could conduct the related interventions. Also, involving stakeholders can provide better access to target populations and add more important local awareness to malaria programmes [21].

There has been tremendous progress in malaria elimination in Iran [23]. The multi-sectoral collaboration of stakeholders in malaria elimination has been considered a vital component of the national strategic plan. Malaria elimination committees, at the provincial and district levels, including members of energy, water supply, agriculture, education, and broadcasting organizations, as well as municipalities and elected community-based councils are chaired by their respective governors. The chancellors of the Medical Sciences Universities are the secretaries at the provincial level. As for the districts, the director of the district’s health network is the correspondent. These committees consult and help decision makers to eliminate malaria. Although, to make full use of all capacities of stakeholders much could still be done [24].

Most of the organizations of malaria-endemic areas of Iran look at the issue of immigrant and refugee population movement as a general issue and are less focused on the potential of malaria transmission. This study intends to draw attention to this aspect of the subject. In this study, the phrase ‘*providing access to services and immigrant and refugee population movement control*’ refers to the process of identifying these populations, providing malaria prevention, diagnosis, treatment and referral services, as well as tracking the border and within-district movements.

There is limited systematic information on the process and outcomes of inter-organizational collaboration to eliminate malaria [25], and even less is known about how to use networks to inform collaborative planning and targeting imported malaria by immigrant and refugee movements [9]. No study has addressed social network analysis in the context of malaria elimination. In addition, social network methods are frequently implemented on egocentric networks [26, 27], while fewer cases have been reported on using whole networks as the unit of analysis [28, 29].

This study aimed to answer the following questions: (1) concerning the access of undocumented immigrants and refugees to malaria services in endemic districts, how do the key stakeholders interact with each other? (2) Which organizations or groups are more central in those collaboration networks? (3) Do these organizations group together and form clusters?

The findings will help the decision-makers plan for improving the level of inter-sectoral collaboration, shaping coalitions, and providing improved access to prevention, diagnosis, and treatment services of malaria among immigrants and refugees through network analysis.

## Methods

The data collection of social networks among stakeholder organizations was carried out in 2016–2017. Social networks are formed by actors/nodes and relational ties, linking nodes together. In the present study, the nodes were stakeholders who provided access of unauthorized immigrants and refugees to malaria services in southeastern Iran. Ties indicated the collaboration quality, which is, in turn, determined by the level of collaboration scale.

### Setting

**Fig. 1:**
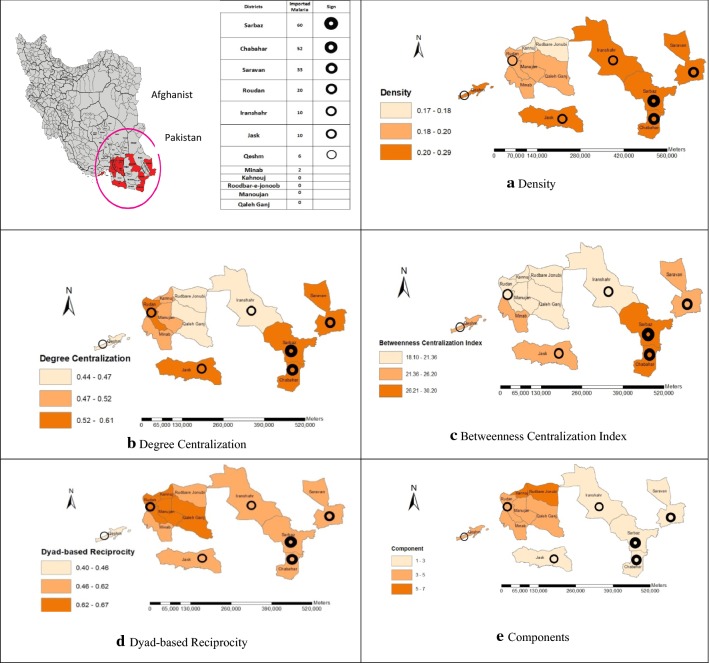
Network analysis measures and imported malaria cases among different Malaria endemic districts of Iran in 2016–2017

The study settings were the malaria-endemic districts in Iran. Iran has participated in the process of eliminating malaria since 2010. In the last few years, almost all malaria incidences have occurred in southeastern Iran and provinces adjacent to the Pakistan border. Imported malaria cases were caused by the movement of immigrants and refugees crossing the Afghanistan and Pakistan borders [30]. For this purpose, 12 districts in Sistan and Baluchestan, Hormozgan, and Kerman provinces were selected (four districts within each province). The selection criterion was readiness declaration from their governors. Figure 1 illustrates the location of these districts.

### Study participants

The list of stakeholders was extracted from interview with malaria programme authorities in malaria-endemic districts and then consulted in a group, consisting of the principal researcher, district’s health centre authorities, representatives of non-governmental organizations (NGOs), private organizations, and the community representative for each district, based on the World Health Organization (WHO) guideline of stakeholders’ analysis [31].

A list of 30 extracted organizations represents the entire list of stakeholders related to immigrants and refugees access to services. One informed representative of each organization was included in the study. If an organization had more than one representative, one was chosen at random. Finally, 360 individuals (30 representatives of the organization/group in each district) were included in the study. Additional file 1 represents the list of organizations/groups with their abbreviation. The roles of organizations/groups concerned controlling immigrant and refugee population movement and providing access to malaria-related services summarized in Box 1.

Box 1: Nature of participating organizations/groups and their role in Malaria elimination *Coordinating Organization*: District Governor.

*Civil Organizations* (District Authority, District Council, Village Authority, Village Council, District Council, City Council, Municipality): Knowledge about districts to identify patterns of immigrant movement, case finding, providing Malaria prevention services.

*Volunteer community representatives* (Trustees/Community Volunteers, Charities, Religious Leaders, Red Crescent, Immigrants, and Mobile Religious Groups): Trust building among residents and immigrants, resource mobilization, volunteers’ involvement, case findings, diagnosis.

*Employment and income generation related organizations* (Trade union, Harbor, Free Zone Organizations, Private Employers, Farmers, Welfare, Fisheries, labor, as well as Agriculture Organizations): Identifying newcomers and illegal workers, selecting representatives among them for case finding and diagnosis.

*Cultural Organizations* (Education and Culture and Islamic Guidance Organizations, Media): Communication, education, persuading the community to be involved in case finding.

*Health Organization*: Providing Malaria prevention, diagnosis and treatment services.

Security Organizations (Foreign Immigrants Affairs, Police, Army, Frontier Control Office Information Organization): Controlling illegal population movement across borders and introducing arrested illegal immigrants to the community health workers- before deporting them- to provide diagnosis and treatment services.

*Illegal Traffic Groups* (Smugglers): Case finding, diagnosis of malaria among illegal immigrants and refugees by using RDT kits which are provided by community health workers.

Data collection The data were collected using levels of collaboration scale of Frey et al. [32]. The validity and reliability of the questionnaire have been tested (end of year 1: α = 0.87) [32]. Further, the researchers conducted forward–backward translation procedures and investigated the face validity of scale between stakeholders, who provided immigrants and refugees access to services, and controlled their movement in the malaria elimination programme, by reviewing the participants’ opinions. The content validity of the scale was determined by 10 experts in the fields of inter-sectoral collaboration, malaria, and health promotion. Test–retest reliability of the scale was confirmed during a 2-week interval.

The questionnaire filled in during interviews had two components: (1) Demographic characteristics of the organization, including title, type (governmental, non-governmental, private, or community based) and demographic characteristics of the respondents, including age, gender, position, history of malaria affliction (implying a more personal connection and concern about the issue, which was expected to increase the likelihood of involvement in the malaria elimination programme), and years of service, and (2) The level of collaboration scale. Further, the questionnaire included titles of the stakeholder organizations/groups related to immigrants and refugees access to malaria services. Levels of collaboration are presented: ‘no interaction’, ‘networking’, ‘cooperation’, ‘coordination’, ‘coalition’, and ‘collaboration’ in columns, which their definitions are given in Box 2.

The stakeholders were asked whether they had any relationship in favour of health and delivering malaria services to immigrants and refugees, if so, it was considered as a tie. In case of illegal immigrants and smugglers, the police and immigration officials carry out their legal duties by arresting them. The collaboration was considered as a tie when the police introduced arrested illegal immigrants -before deporting them- to the community health workers to provide diagnosis and treatment services with health benefits in terms of preventing the spread of the disease in the community and among immigrants. It was requested from the representative of each organization/group to identify the relationship level between their organization/group and other stakeholder organizations/groups mentioned in the respective row and column, according to the given definition of collaboration level and relevant examples. A score ranging from 0 to 5 (0: no interaction, 1: networking, 2: cooperation, 3: coordination, 4: coalition, and 5: collaboration) was considered to identify the level of collaboration. The process of data collection was coordinated by the Ministry of Health and Medical Education (MOHME). The objectives of the plan were explained to the participants and their consents to participate were obtained beforehand. The interview was conducted by the main investigator, which lasted an average of 15 min. Names of participants were not requested and people were only interviewed as representatives of the organization/group.

The completed questionnaires were revised and incidental blanks were filled in through contacting the respective person later on. All 360 individuals representing the stakeholders’ list participated in interviews with no missing values.

**Box 2: Levels of collaboration**

0. *No Interaction*: Not aware of this organization, not currently involved in any way.

1. *Networking:* Aware of organization, loosely defined roles, little communication, all decisions are made independently.

2. *Cooperation:* Provide information to each other, somewhat defined roles, formal communication, all decisions are made independently.

3. *Coordination*: Share information and resources, defined roles, frequent communication, some shared decision making.

4. *Coalition*: Share ideas, share resources, frequent and prioritized communication, all members have a vote in decision making.

5. *Collaboration*: Members belong to one system, frequent communication is characterized by mutual trust, and consensus is reached on all decisions.

Data analysis UCINET 6 program was used to analyse the network data. Twelve separate networks were created representing 12 districts. The relationships were transformed into *n* by *n* matrices, in which the rows and columns contained the list of stakeholders, while the strength or the level of collaboration was the value of cells. Collaboration level values greater than or equal to 1 was defined as a tie or collaboration. Measures of density, centralization, reciprocity, and clustering were calculated for each network and at each level of collaboration, as defined in Box 3. The network was symmetrized to calculate the mentioned indices except for the reciprocity.

For each stakeholder in each network, the centrality measures were calculated. The method used by Valente et al. [9] was applied to interpret the obtained scores. The density scores below 0.30, between 0.30 to 0.50, and higher levels of 0.50 were considered low, medium (favourable), and high, respectively. The value less than 0.25 for centralization, more than 0.50 for reciprocity, more than 1 for degree and zero for components were considered as desirable values [38]. The NetDraw program (version 2.141) was used to draw maps and visualize the findings. Further, the ArcGIS software was used to implement indicators on district maps and compare them in terms of the number of imported Malaria cases from March 2016 to February 2017. Correlations of imported malaria cases and network indices were assessed by using Spearman correlation test in Stata software (version 15).

**Box 3: Definitions of network analysis indices**

*Density* means the extent of connectedness in the network, calculated as the number of actual ties divided by the number of possible ties [33, 34].

*Centrality* is an indicator of prominent actors in the network [20]. Degree centrality and betweenness centrality measure two different aspects of prominence:

*Degree centrality* is simply the number of connections each organization has [20].

*Betweenness centrality* is calculated by the number of times an organization mediates the indirect paths between pairs of other organizations, which themselves are not connected to each other directly [20]. Higher betweenness centrality implies greater potentials for brokerage [34].

*Degree centralization* indicates the degree to which the network represents a star-shaped network with one central actor to which all other actors are connected [35].

*Betweenness centralization* demonstrates the extent to which the distribution of betweenness centrality represents a star-shaped network or the degree to which one organization is able to control the relationships with other related organizations in the network [35].

*Reciprocity* measures the proportion of mutual (bi-directional) connections [26].

*Clustering* means the extent to which nodes are classified by an attribute [34]. Clustering coefficient (CC) is a measure of cohesion, which is calculated by measuring the density of each node’s ego network (partners) [36].

In this study, CC was the density among all other stakeholders maintaining a direct connection to each respondent.

A part of the network by which all actors are directly or indirectly related to at least one tie is called ‘*connected component*’. In this regard, each isolate is considered as a separate component [20]. *Clique* is regarded as a subgroup of actors who are all directly connected to each other [20, 33].

In a *core*-*periphery analysis*, core nodes are recognized as densely connected and peripheral nodes are considered as sparsely connected nodes [37].

### Ethics approval

Ethical criteria have been approved by the Ethics Committee of Tehran University of Medical Sciences (code no. 8921108003).

## Results

### Demographic characteristics

The average age of the participants was 46 (SD = 8.4). Most of them came from governmental organizations (52.8%) and held a chief position (49.4%). About 94.4% were male. The majority of the participants (43.9%) had more than 10 years of service. As for a history of malaria affliction, 33% showed a positive history. Table 1 presents the demographic characteristics of the participants and their organizations/groups.

**Table 1:** Demographic characteristics of study participants

Variables	Categories	Number (percent)
Type of organization (n = 360)	Government	190 (52.8)
	Community based organization	111 (30.8)
	Non-governmental organization	22 (6.1)
	Private	37 (10.4)
Position (n = 360)	Chief	178 (49.4)
	Assistant	105 (29.2)
	Community representatives	77 (21.4)
Years of service (n = 310), years	1–3	29 (9.3)
	3–5	52 (16.6)
	5–10	94 (30.1)
	> 10	137 (43.9)
History of malaria affliction (n = 358)	Yes	33 (9.2)
	No	325 (90.8)
Sex (n = 360)	Male	340 (94.4)
	Female	20 (5.6)

### Network characteristics

#### Density

The mean networks density across all 12 networks was 22% (Mean ± SD: 0.22 ± 0.04) and the average of total ties across networks was 198. The values of network density in different districts are presented in Table 2. Network density was higher in districts with a common border (land or sea) with other countries like Chabahar, Saravan, Jask, and Sarbaz, by 29, 27, 27 and 24%, respectively.

**Table 2:** Networks analysis measures in Malaria endemic districts of Iran in 2016–2017

No	Districts	Density	No. of relations	Avg. degree	Degree centralization	Betweenness Centralization Index	Dyad-based reciprocity
1	Chabahar	0.29	126	8.4	0.61	0.3	0.57
2	Iranshahr	0.24	102	6.8	0.45	0.21	0.6
3	Saravan	0.27	119	7.93	0.55	0.23	0.6
4	Sarbaz	0.24	108	7.2	0.54	0.28	0.6
5	Jask	0.27	120	8	0.59	0.24	0.55
6	Qeshm	0.24	107	7.13	0.47	0.26	0.46
7	Minab	0.19	85	5.66	0.52	0.24	0.62
8	Roudan	0.2	88	5.86	0.55	0.27	0.67
9	Kahnouj	0.18	82	5.46	0.5	0.18	0.64
10	Rudbar-e-Jonoob	0.17	76	5.06	0.44	0.19	0.6
11	Manoujan	0.2	89	5.93	0.48	0.19	0.64
12	QalehGanj	0.19	86	5.73	0.45	0.19	0.67

Figure 1 illustrates that higher network density in districts is associated with higher incidence of imported malaria. There was a significant correlation between the density of networks and imported malaria cases (r = 0.66, P = 0.02). As shown in Table 3, by increasing the level of collaboration from level 1 (networking) to level 4 (coalition) among stakeholders in 11 out of 12 districts (91.66%), the network densities have decreased. However, there is a downtrend in Jask district, where there is a slight increase in the density index from level 1 (networking) to level 2 (cooperation). In all the surveyed districts, an average %10 of the reported relationships was in networking (Mean ± SD: 0.10 ± 0.02), 8% in cooperation (Mean ± SD: 0.08 ± 0.01), 4% in coordination (Mean ± SD: 0.04 ± 0), and 0% in coalition levels of collaboration. The remaining 78% did not have any interaction with other organizations (Mean ± SD: 0.78 ± 0.04).

**Table 3:** Networks analysis measures according to levels of collaboration among Malaria endemic districts of Iran in 2016–2017

No	Districts	Indicators	Levels of collaboration			
			Coalition	Coordination	Cooperation	Networking
1	Chabahar	Density	0.12	0.1	0.05	0
		Degree^a^	0.23	0.29	0.16	0
		Betweenness^b^	25.5	29	6.99	0.12
		Reciprocity	0.21	0.36	0.57	0.33
2	Iranshahr	Density	0.12	0.1	0.05	0
		Degree	0.14	0.31	0.14	0
		Betweenness	16.1	18.1	1.75	0.12
		Reciprocity	0.17	0.42	0.64	0.5
3	Saravan	Density	0.12	0.07	0.06	0
		Degree	0.19	0.25	0.26	0.1
		Betweenness	18.6	12.8	11.2	0.12
		Reciprocity	0.21	0.35	0.56	0.25
4	Sarbaz	Density	0.11	0.07	0.05	0
		Degree	0.17	0.35	0.16	0.06
		Betweenness	12.4	23	4.61	0.12
		Reciprocity	0.27	0.39	0.45	0.33
5	Jask	Density	0.09	0.12	0.05	0
		Degree	0.19	0.42	0.19	0.06
		Betweenness	12.6	23.7	10.7	0.12
		Reciprocity	0.2	0.29	0.59	0.33
6	Qeshm	Density	0.11	0.08	0.04	0
		Degree	0.24	0.2	0.14	0.06
		Betweenness	12.4	14.8	4.23	0.12
		Reciprocity	0.24	0.3	0.38	0.5
7	Minab	Density	0.08	0.07	0.03	0
		Degree	0.2	0.35	0.14	0.03
		Betweenness	11.9	20.2	1.24	0
		Reciprocity	0.26	0.48	0.66	1
8	Roudan	Density	0.09	0.06	0.04	0
		Degree	0.19	0.3	0.17	0.03
		Betweenness	11.6	16.4	5.53	0
		Reciprocity	0.29	0.5	0.8	1
9	Kahnouj	Density	0.07	0.06	0.04	0
		Degree	0.17	0.26	0.17	0.06
		Betweenness	9.88	13.2	1.78	0.12
		Reciprocity	0.3	0.42	0.66	0.5
10	Rudbar-e- Jonoob	Density	0.06	0.07	0.03	0
		Degree	0.15	0.29	0.14	0.03
		Betweenness	12.7	8.95	9.3	0
		Reciprocity	0.18	0.48	0.68	1
11	Manoujan	Density	0.09	0.06	0.04	0
		Degree	0.19	0.26	0.2	0.06
		Betweenness	15.3	16.3	3.05	0.23
		Reciprocity	0.22	0.37	0.59	0.5
12	QalehGanj	Density	0.08	0.06	0.04	0
		Degree	0.17	0.28	0.16	0.06
		Betweenness	12.2	15.1	7.82	0.12
		Reciprocity	0.24	0.38	0.63	0.33

^a^Degree centralization

^b^Betweenness network centralization index

### Centrality

The results of examining degree and betweenness measures are presented below:

*Degree centralization* The average degree centralization of networks was 0.51 (SD: 0.05). The corresponding values of different districts are listed in Table 2, which is at a high level in Chabahar and Qeshm. Figure 1b represents a higher level of degree centralization in districts with more imported malaria incidences. Further, there was a significant correlation between degree centralization of networks and imported malaria cases (r = 0.65, P = 0.02).

*Degree centrality* In reviewing the stakeholder’s network in different districts, there were no considerable differences in the distribution of degree centrality among stakeholders. Five stakeholders with the highest degree centrality in all districts were HLT (Health), DG (District Governor), FIA (Foreign Immigrants Affairs Organization), DA (District Authority), and TRT (Trustees/Community Volunteers), respectively.

Table 4 presents the list of key stakeholders maintaining the most collaboration with other stakeholders and with a higher level of degree centrality.

**Table 4:** Ranking stakeholders according to levels of degree centrality and betweenness centrality in 2016–2017

Rank	Stakeholders	Degree Mean (SD)	Stakeholders	Betweenness centrality Mean (SD)
1	HLT	0.65 (0.10)	HLT	22.57 (5.86)
2	DG	0.63 (0.07)	DG	19.58 (3.51)
3	FIA	0.47 (0.09)	FIA	4.22 (1.48)
4	DA	0.43 (0.08)	DA	3.58 (2.51)
5	TRT	0.38 (0.03)	TRT/FSR	2.84 (2.10)
6	ARM	0.36 (0.05)	EDU	2.22 (1.14)
7	VA	0.31 (0.04)	RL	1.47 (1.06)
8	PLC	0.30 (0.03)	LBR	1.29 (0.95)
9	DC/RL	0.23 (0.03)	VA	1.12 (0.45)
10	SC/VC	0.23 (0.04)	ARM	1.10 (0.35)
	Total mean (SD)	0.22 (0.04)	Total mean (SD)	2.26 (0.95)

Additional file 2 maps stakeholder networks which provided access of immigrants and refugees to malaria services and/or controlled these population movements in order to eliminate malaria, and the degree centrality of various organizations/groups in the studied districts. In each map, a node represents an organization/group in the network and the tie between two nodes reflects the level of collaboration between stakeholders. The size of the network in each district is 30. An increase in the square size indicated a rise in the centrality of relevant organizations/groups. The lines’ thickness represents an increase in the level of collaboration. Weak and strong ties in the collaborative networks are visible in maps.

By increasing the level of collaboration in 11 districts, the degree centralization index rose from level 1 (Networking) to level 2 (collaboration) and declined at levels 3 (coordination) and 4 (coalition) (Table 3).

In general, degree centralization of reported relationships was 0.18 at the networking level (SD: 0.02), 0.29 at cooperation level (SD: 0.05), 0.17 at coordination (SD: 0.03), and 0.05 at coalition level (SD: 0.02). Figure 1b displays the degree centralization indices in various districts and network connections among stakeholders related to immigrants and refugees access to malaria services.

*Betweenness centralization* The average betweenness centralization of the networks was 0.23 (SD: 3.93). Betweenness centralization was relatively higher in Sarbaz and Chabahar. Figure 1c illustrates that higher betweenness centralization of the networks in districts is associated with greater imported malaria incidence. There was a significant correlation between betweenness centralization of networks and imported malaria cases (r = 0.76, P = 0.004).

*Betweenness centrality* Five stakeholders with the highest betweenness centrality were HLT, DG, FIA, TRT, and FSR (Fisheries organization), respectively (Table 4).

### Reciprocity

In general, the proportion of mutual interactions was 0.60 (SD: 0.05). Figure 1d represents the higher reciprocity which was not related to regions with more imported malaria cases. As can be seen in Table 3, reciprocal connections increased as the level of collaboration between stakeholders rose from level 1 (Networking) to level 3 (Coordination). In general, an average 0.23 (SD: 0.04) of the reciprocal relationships was at the networking level, 0.39 at the cooperation level (SD: 0.06), 0.60 at the coordination level (SD: 0.10), and 0.54 at the coalition level (SD: 0.28).

### Clustering

The average clustering coefficient was 0.54 (SD: 0.03). As Fig. 1e shows, districts with high imported malaria cases have fewer components (Chabahar and Saravan:1, Jask:2, Iranshahr and Sarbaz:3, Qeshm:4, Minab and Roudan:5, Manoujan and Qaleh Ganj:5, and Kahnouj and Rudbar-e-Jonoob:7). The isolated groups included MDA (Media), WLF (Welfare Organization), CIG (Culture And Islamic Guidance Organization), CRT (Charity), AGR (Agriculture Organization), RC (Red Crescent Organization), and NGOs. In terms of the number of cliques in networks, the results indicated that there was an increased tendency to create cliques in districts with high imported malaria cases (Chabahar, Iranshahr, Saravan, Jask, Gheshm, and Sarbaz by 41, 38, 36, 35, 33 and 32, respectively). The lowest cliques belonged to Rudbar-e-Jonoob (20 cliques).

In most networks, the HLT, DG, FIA, PLC (Police), DA, VA (Village Authority), TRT, IM (Immigrants and Refugees Representatives), ARM (Army), FSR, FC (Frontier Control Office) and INF (Information Organization) group, were in the core with an average density of 0.80 (SD: 0.05). These central groups, in association with periphery groups, had an average density of 0.39 (SD: 0.03), which is moderate. Further, the periphery group had a weaker relationship with the internal periphery organizations/groups, with an average density of 0.21 (SD: 0.02).

## Discussion

Collaborative networks play a significant role in the design, implementation, and sustainability of successful programmes, including malaria elimination programmes [7, 39]. The present study aimed to investigate the structure of inter-sectoral collaboration networks related to immigrants and refugees access to services in order to eliminate malaria. Results indicated that the average network density in districts was %22. Although some sources have considered density over %15 and below %50 as favourable [38, 40], according to the criteria considered in the study, it is relatively low. At a low-level density, distinct subgroups will be generated within the network, that can have a negative impact on subgroup collaboration capacity [41].

The network density is higher in border districts and districts with more imported malaria cases, which may indicate higher preparedness against the issue and the formation of a large number of relationships in these districts for controlling immigrant movements. In this regard, Feinberg et al. [42] found that network density is positively related to the community’s readiness to engage in community-based coalitions and diversity of involved organizations, facilitate this matter.

The results revealed that the highest density is present at the lowest level of collaboration or networking. Strategies such as identifying common goals and mission can help improve network connections at the higher levels of collaboration [38], especially relationships among stakeholders can help decrease malaria cases and increase access of immigrants and refugees to services. A critical review of the literature by Smith et al, on malaria and population mobility, suggested that the collaboration of stakeholders especially in border areas and regions of high population mobility and imported malaria is important to achieve malaria elimination goals. A collaborative approach would better equip malaria elimination programmes to access populations through social networks and to identify other potential sites of transmission, at which they could carry out interventions [15].

The second question focuses on whether some organizations/groups are more crucial due to their position in the network. Many programmes have used random, inaccurate, and available methods to identify the actors who can play a more effective role in implementing and delivering programmes and facilitating access to resources [43]. These influential stakeholders can assist in recognizing the needs of community, obstacles, and incentives for behaviour change [9].

The following paragraphs focus on identifying central network actors as potentially prominent and influential members of the community, as well as isolates (actors with no connections) as potentials for strengthening collaboration. Results of centrality measures of symmetrized network indicated that the HLT and DG are the most active stakeholders that can potentially have an impact on the access of immigrants and refugees to health services as key determinant of malaria elimination, due to their intermediary role between organizations (higher betweenness centrality) and ability to build more connections (higher degree centrality). In terms of degree centrality and betweenness centrality, FIA, DA, and TRT/community volunteers stand out in the subsequent category as the most active in communicating with other stakeholders of the network and may have the potential impact on decision-making in controlling immigrant and refugee movements. The stakeholders with the highest centrality, especially DG, FIA, and DA have political delegated power from provincial and national levels to make decisions related to the district and mobilize organizations [44]. Studies have also shown the power of TRT in the traditional context of target areas in mobilizing the community, increasing their awareness, and building trust which affects engagement with malaria elimination authorities and shaping health-seeking behaviours. Furthermore, a partnership with trustees allows health programme to reach vulnerable and marginalized communities. They can work as a strong link between the healthcare system and these communities [45, 46]. Studies have found that social networks have a positive influence on health-seeking behaviours including what health services to access, and when, where and how frequent to access them. Social networks can be employed as a channel for dissemination of health information and addressing the cultural factors that influence health information acquisition and access to services for immigrants and ethnic minorities [47, 48]. Malaria elimination programme can successfully use social networks to communicate with unregistered workers, and recruit peers of target groups to be as community health volunteers and gain their trust [15]. Based on the results, collaborative interventions of malaria elimination programme may benefit from pairing members with high and low degree centrality in smaller working groups, as suggested by other programmes [38].

The results of this study, in terms of degree centrality and betweenness centrality measures, demonstrate the role of trusted local community leaders in communicating with other stakeholders of the Malaria elimination programme. Their collaboration in the network can be helpful in the selection of interventions, adapting intervention strategies to local conditions, implementation, and evaluation to change the behaviour of the community [9]. The significance of the community leaders has also been emphasized in other studies [17]. They act as gatekeepers and can control the flow of resources among stakeholders who are connected. Other stakeholders can benefit from these intermediary points that are in or along the path of many other stakeholders and have the power to restrict or promote communications [20, 41]. Due to the intermediary role of some organizations (HLT, DG, FIA, and TRT), advocacy and dialogue with gatekeepers can be helpful in introducing stakeholders to each other and removing obstacles to facilitate partnership in order to eliminate malaria.

In this study, the average networks’ degree centralization is 0.51, which is high. This reflects that the network tends to be affected by one or more specific organizations [35]. Results show the degree centralization has been roughly at a high level in border districts and districts with more imported malaria cases, which probably indicates the dominance of several organizations and centralization of power in decision-making in the network to help a better top-down outbreak management. It seems that the network naturally evolved into a hierarchical order in response to the increasing cases near the borders, to provide better solutions for the problem. It is not unusual to respond to infectious disease outbreaks with incident command system, which provides hierarchy so that multiple agencies coordinate effectively to reduce the threat [49]. By increasing the level of collaboration to cooperation, the network is affected by certain organizations. However, decentralization of power has occurred at higher levels of collaboration. In this regard, Feinberg et al. have realized that there is a negative correlation between increasing network centralization and community readiness to engage in coalitions, hence preventing a collective action [9, 42]. Higher levels of centralization of power among a limited number of stakeholders (actors) can create challenges for participatory management and sharing the power among all individuals [50].

It may be helpful to conduct interventions to reduce the centrality of specific organizations in malaria programme, by empowering and engaging with other marginalized organizations in the decision-making process. The results showed that the average betweenness centralization index of networks was %22, which is relatively low, reflecting the higher number of gatekeepers [35].

Furthermore, betweenness centralization of networks in border districts with more imported malaria cases is at a high level. It seems that in these areas, where the cases are higher, the network has evolved into a compartmentalized one to provide a better response to the situation. In networks as such, a number of intermediary stakeholders may establish connections with other subgroups. With the collaboration rising to the cooperation level, one or more organizations assumed greater control over the relationship between stakeholders, but at the higher levels of collaboration, this control has been decreased. The results represent distribution of mediation role among a wider range of stakeholders and at higher levels of collaboration.

The third question of this study focuses on reciprocity. The results showed that more than half of the networks’ connections are generally two-way and desirable. Although the density of the networks is relatively low, the existing relationships are more bilateral, which can be a sign of network sustainability and resilience during the crisis [51], trust, coherence, social capital [52], sharing interests, exchanging resources, and emotional support [53] or even social and legal obligations. Reciprocity of relationships is not related to the number of imported malaria cases, except for districts of Kerman province. Perhaps the culture of cooperation in the region plays a role in the establishment and maintenance of bilateral relations between organizations. Based on the results, reciprocity of relations rose by increasing the level of collaboration between the stakeholders.

Relationships among pairs of stakeholders are unique and context-dependent. We have explored the contextual dynamics among the stakeholders in a qualitative study titled “Exploring components of an advocacy programme for inter-sectoral collaboration in the elimination of malaria” which is in the publication process. Audiences and their interests of participation, messages as well as channels of communication were explored. Regarding the local conditions of the study, existing reciprocal interactions between stakeholders can be considered as an indicator of trust. When the stakeholders had trust together, the relationships in favor of health and delivering malaria services to immigrants and refugees were shaped. Regarding qualitative study results, people who are living in two sides of the border have similar ethnic, religious, linguistic, and cultural characteristics. These people have familial, marital, and commercial relationships with each other. For these reasons, immigrants who are trafficked into the country, mainly for occupational reasons, are partially accepted and welcomed in these areas. Community health workers and trusted health volunteers in these areas, who are from indigenous people, interact with some of the traffickers and provide them with a free rapid diagnostic test to help malaria diagnosis. Traffickers’ collaboration is based on trust and for the benefit of their own health and of the residents. In order to improve mutual interaction between stakeholders who maintain a one-way relationship, they can be grouped together in collective activities [38].

The fourth question focuses on the subgroups of the network. The results showed that the clustering coefficient was 0.54, which is relatively high. This value represents higher willingness of stakeholders to cluster together in the network. Districts with high incidences of imported malaria had fewer components and those with fewer incidences had more connected components. It seems that high sensitivity to the issue in the networks of near border areas, may lead organizations to participate and have better communications with each other. In districts with fewer imported malaria cases, stakeholders who have not been involved and are isolated have caused a disconnection. Interventions that can be defined in the malaria elimination programmes should try to reduce these subgroups, fit them into small workgroups and bridge the gap between them so that any stakeholder can communicate with others, both directly and indirectly [38].

Isolated stakeholders include MDA, CIG, WLF, CRT, AGR, RCs, and NGOs. Access to information, resources, and services in the network is difficult for isolated organizations/groups. Involving these stakeholders and expanding network links can assist their empowerment and improve intersectoral collaboration [9]. Interventions in malaria elimination programme can prevent isolated stakeholders from disappearing in the background by paying specific attention to these groups through separate meetings and peruse their concerns about the network objectives and linking them to smaller groups, with members who have high levels of connection with others [38]. The expansion of network links to the media can be effective as well since interventions that use the media in the social networks are effective and can reinforce behavioural change messages and provide feedback from stakeholders on the proposed strategies [9, 54].

Furthermore, the role of the agricultural organization should not be overlooked in facilitating access to immigrants and refugees, who are mostly engaged in seasonal work, also in this category are nongovernmental organizations, welfare organization, and charities providing malaria services to marginalized immigrant communities, and culture and Islamic guidance organization delivering educational and cultural services.

The HLT, DG, FIA, PLC, DA, VA, TRT, IM, ARM, FSR, FC, and INF groups are in the centre and have high density in most networks. Most organizations in the centre are affiliated to the government. This indicates the need for involving private, non-governmental organizations and representatives of immigrant and refugee groups. The review of papers on the use of participatory methods within communicable disease control programmes over 60 years demonstrates that community and collaboration of the vulnerable individuals have played an important role in many successful communicable disease control programmes and increased access to malaria-related services [17, 55].

There is a recall bias in social network analysis, as it usually uses self-report information about existing relationships. However, in the present study, a list of stakeholders was made available to individuals (using roster), which increased reliability. One of the limitations of the present study is the survey of individuals’ perception of collaboration and not the study of existing evidence for collaboration. Another limitation is the inability to distinguish between personal attitudes of individuals and their attitude as the organization’s representative towards the organization’s relations with other stakeholders. This study attempted to overcome this challenge with an emphasis on replying to interview questions as an organization’s representative.

## Conclusion

Social network analysis can help comprehend the complex relationships among organizations and their roles. Higher values of network indices in border districts and districts with more imported malaria cases, in terms of the respective density and centralization measures, may propose the hypothesis that the dominance and centralization of power of several organizations can help a better top-down outbreak management and increase the role of intermediary organizations to connect different subgroup; this area needs further investigations. This study emphasizes the importance of the roles shouldered by local community leaders and authorities in communicating with other stakeholders in the malaria elimination programme, which requires capacity building to participate in deciding what interventions to select, their implementation, and the evaluation of malaria elimination programme. Many organizations, including non-governmental, charity and private organization/groups, are out of the collaboration arena, with respect to the malaria elimination programme; decision-makers are required to devise strategies to effectively engage these groups.

Even though social network analysis considers network actors and their relationships as homogeneous nodes and ties, it is crucial to consider that each stakeholder and the relationship among pairs of stakeholders are unique and context-dependent. It is important to carefully consider the nature of stakeholders, the power relationships between stakeholders, and the formal and informal cultural and contextual factors. Social network analysis is an important method for measuring the degrees of inter-connectedness, however further local knowledge of the context is essential in deciding which nodes within the social network are the most relevant and supportive partners for malaria elimination. Recognition of the existing network structure can help the authorities increase access to malaria prevention, diagnosis, and treatment services among immigrants and refugees.

## Additional files

**Additional file 1.** List of stakeholders and abbreviations.

**Additional file 2.** Maps of stakeholders’ networks related to immigrants’ and refugees’ access to services and control their movement in the field of malaria elimination among endemic districts of Iran in 2016–2017.

## Abbreviations

SNA: Social network analysis
WHO: World Health Organization
MOHME: Ministry of Health and Medical Education
HA: Health Authority
DG: District Governor
FIA: Foreign Immigrants Affairs organization
PLC: Police
DA: District Authority
DC: District Council
VA: Village Authority
CC: City Council
MDA: Media
PRV: Private organization
LO: Labour organization
TRT: Trustees/community volunteers
AGR: Agriculture organization
PAR: Parliament representative
EDU: Education organization
SML: Smugglers
TU: Trade union
CRT: Charity
SC: Security council
MCP: Municipality
ARM: Army
VC: Village Council
IM: Immigrants
FSR: Fisheries organization
RL: Religious leaders
RG: Mobile Religious Groups
HRB: Harbor organization
FC: Frontier Control Office
FZ: Free zone
WLF: Welfare Organization
CIG: Culture and Islamic Guidance Organization
RC: Red Crescent Organization
INF: Information organization
