# Supplementary material for: A social network analysis on immigrants and refugees access to services in the malaria elimination context
Source: Malar J. 2019 Jan 3;18:1. doi: 10.1186/s12936-018-2635-4 (PMC6317246; doi:10.1186/s12936-018-2635-4)
Supplement: Supplementary file 1 — Additional file 1. List of stakeholders and abbreviations. [file 12936_2018_2635_MOESM1_ESM.doc]

**Additional file 1: List of stakeholders and abbreviations**

| **Abbreviation** | **Organization/Groups** | **No** |
| --- | --- | --- |
| **HA** | Health Authority |  |
| **DG** | District Governor |  |
| **FIA** | Foreign Immigrants Affairs org. |  |
| **PLC** | Police |  |
| **DA** | District Authority |  |
| **DC** | District Council |  |
| **VA** | Village Authority |  |
| **CC** | District Council |  |
| **MDA** | Media |  |
| **PRV** | Private Org. |  |
| **LBR** | Labor Org. |  |
| **TRT** | Trustees/Community Volunteers |  |
| **AGR** | Agriculture Org. |  |
| **PAR** | Parliament Representative |  |
| **EDU** | Education Org. |  |
| **SML** | Smugglers |  |
| **TU** | Trade union |  |
| **CRT** | Charity |  |
| **SC** | Security Council |  |
| **MCP** | Municipality |  |
| **ARM** | Army |  |
| **VC** | Village Council |  |
| **IM** | Immigrants |  |
| **FSR** | Fisheries Org. |  |
| **RL** | Religious Leaders |  |
| **RG** | Mobile Religious Groups(Tablighi Jamaat)* |  |
| **HRB** | Harbor Org. |  |
| **FC** | Frontier Control Off. |  |
| **FZ** | Free Zone |  |
| **WLF** | Welfare Org. |  |
| **CIG** | Culture And Islamic Guidance Org. ** |  |
| **RC** | Red Crescent Org. |  |
| **INF** | Information Org. |  |

***** The Outreach society is an ideological movement of Sunni Muslims that has no political affiliation. Members go to various villages or neighborhoods to preach the word of God (Allah).

******  licenses for the production of media are required to be obtained from the Culture and Islamic Guidance Org. Proposing of policies and guiding principles of cultural and educational activities is related to this organization.
