# Supplementary material for: A social network analysis on immigrants and refugees access to services in the malaria elimination context
Source: Malar J. 2019 Jan 3;18:1. doi: 10.1186/s12936-018-2635-4 (PMC6317246; doi:10.1186/s12936-018-2635-4)
Supplement: Supplementary file 2 — Additional file 2. Maps of stakeholders’ networks related to immigrants’ and refugees’ access to services and control their movement in the field of malaria elimination among endemic districts of Iran in 2016–2017. [file 12936_2018_2635_MOESM2_ESM.doc]

| 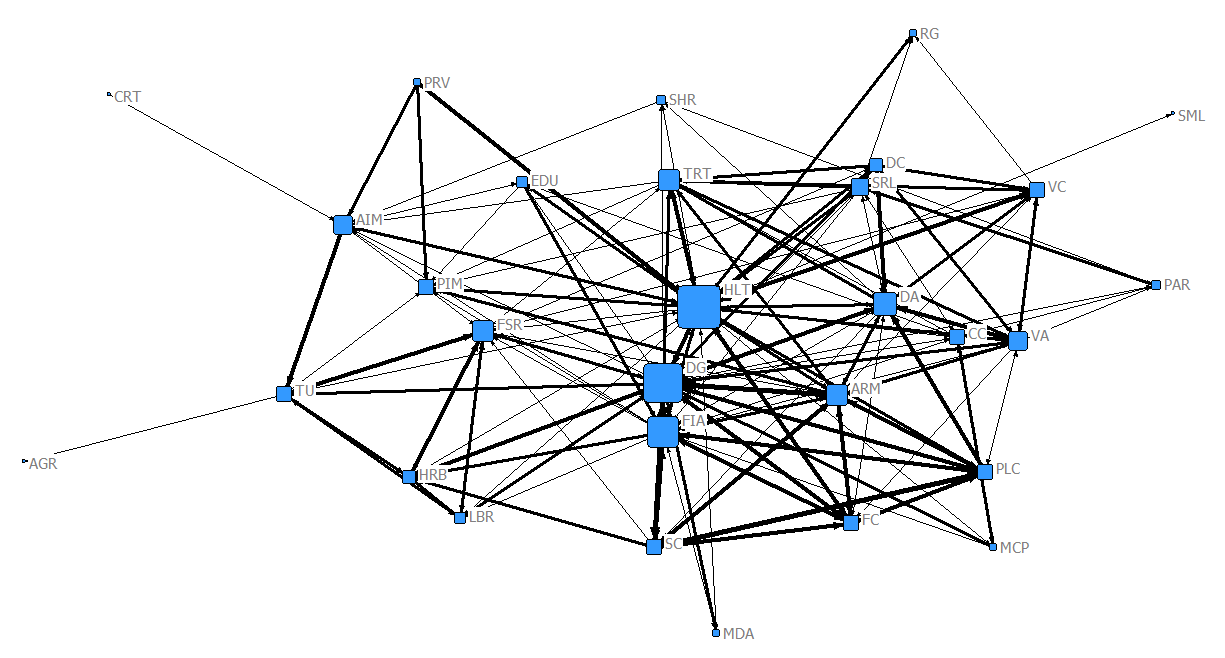 | 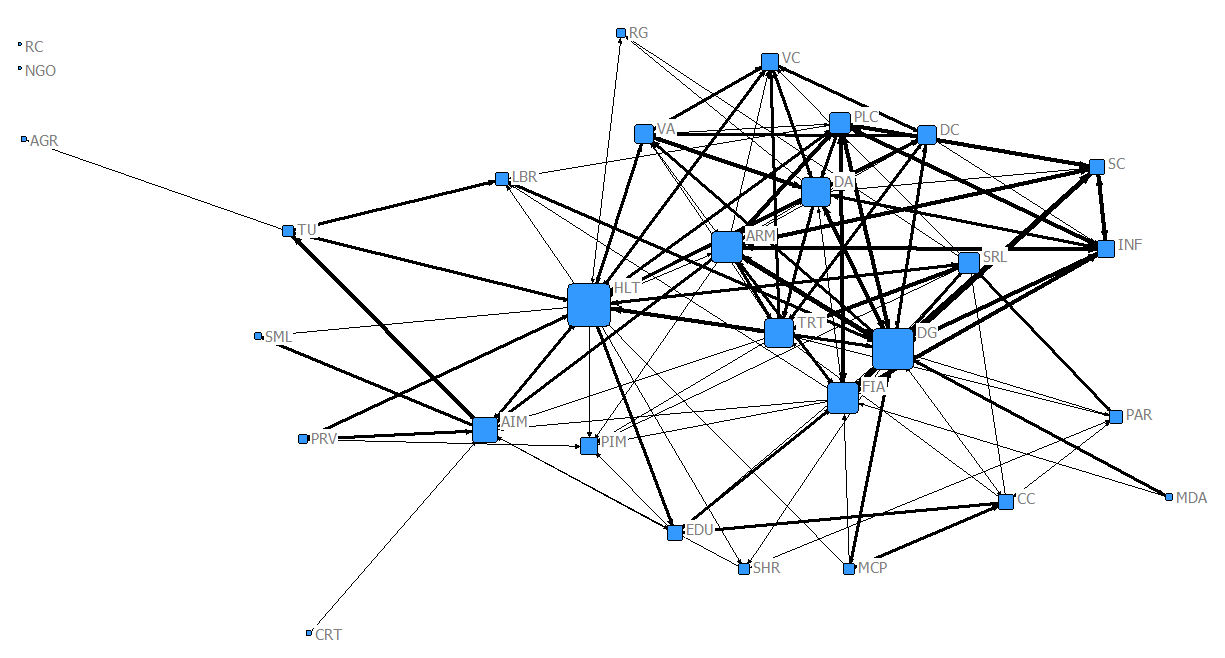 |
| --- | --- |
| Chabahar District  Isolate: 0 | Iranshahr District  Isolate:RC, NGO |
| 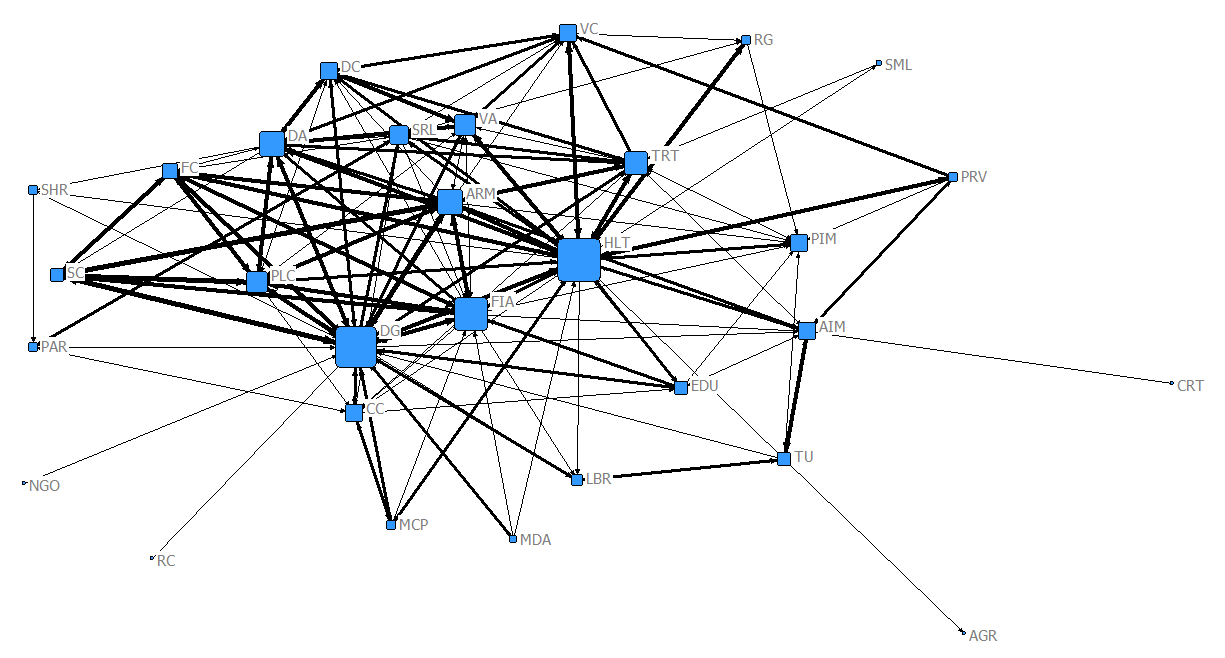 | 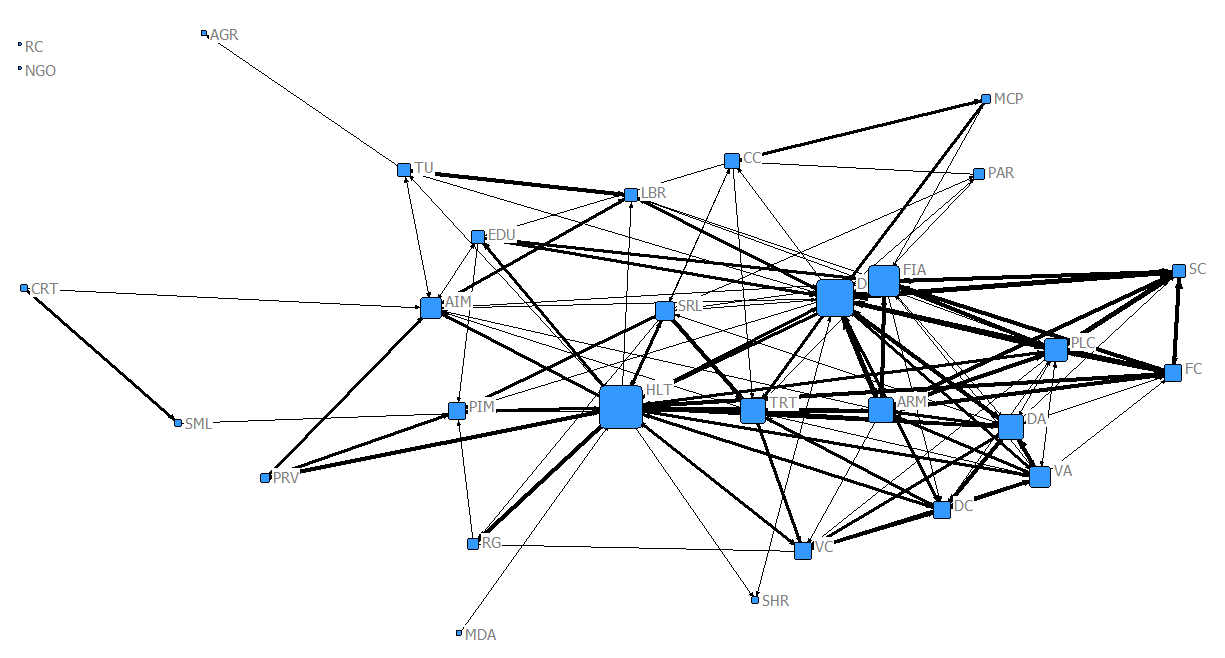 |
| Saravan District  Isolate: 0 | Sarbaz District  Isolate: NGO,RC |

| 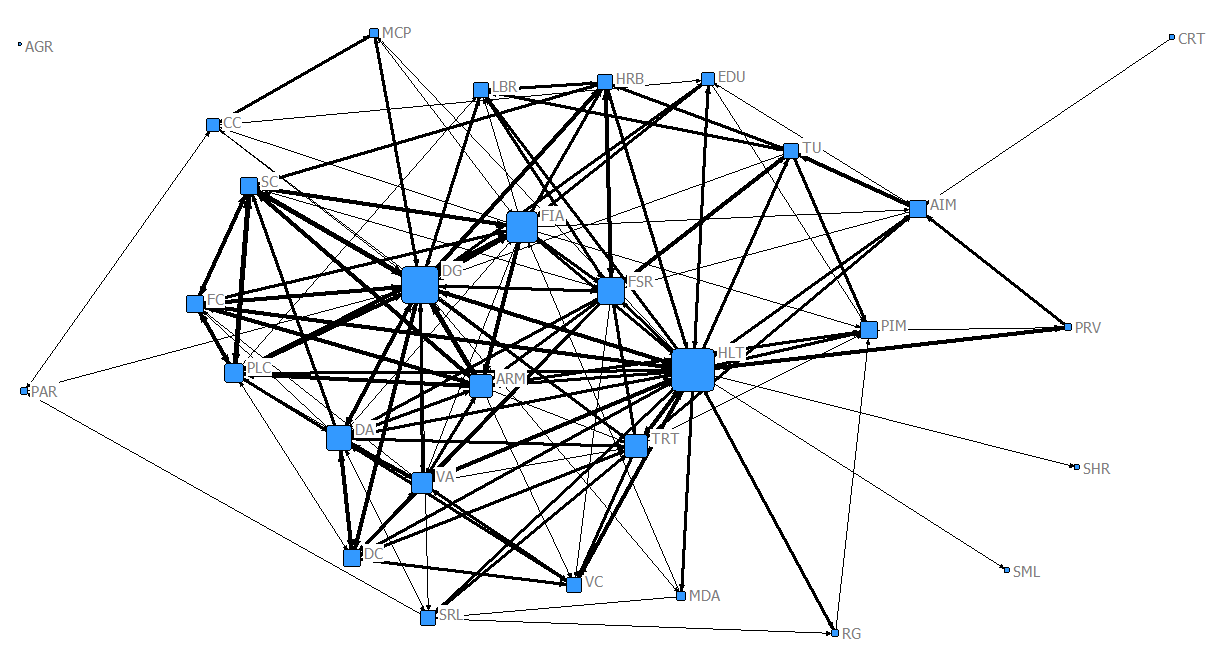 | 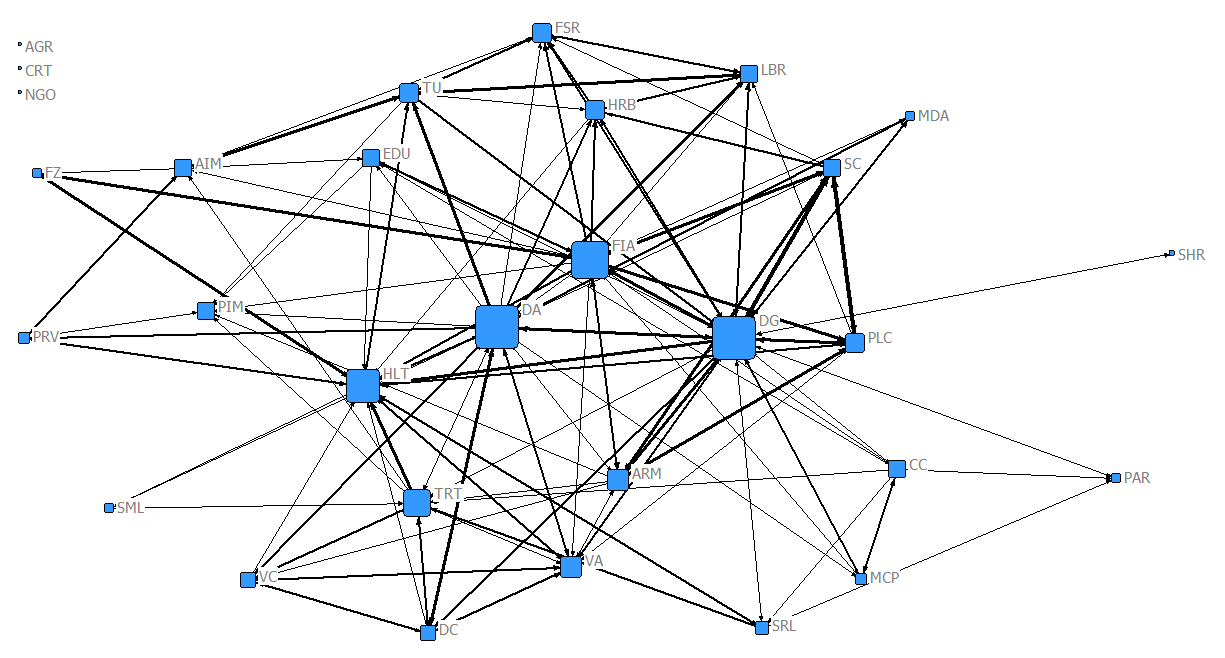 |
| --- | --- |
| Jask District  Isolate: AGR | Qeshm District  Isolate: NGO,CRT,AGR |
| 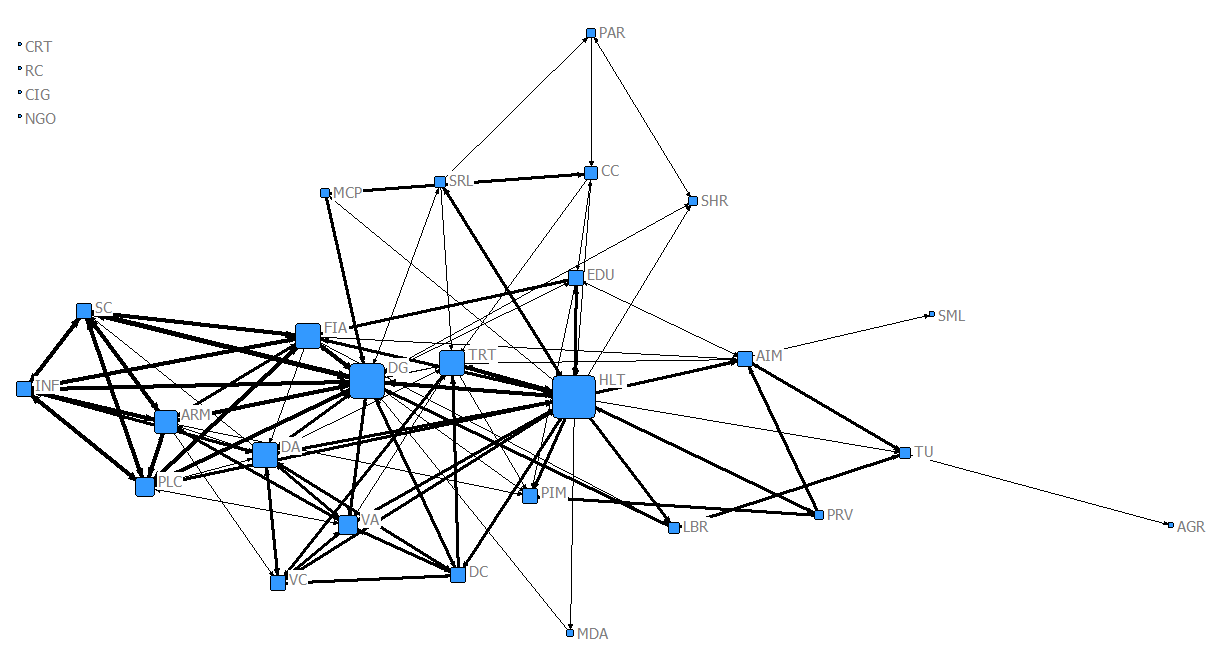 | 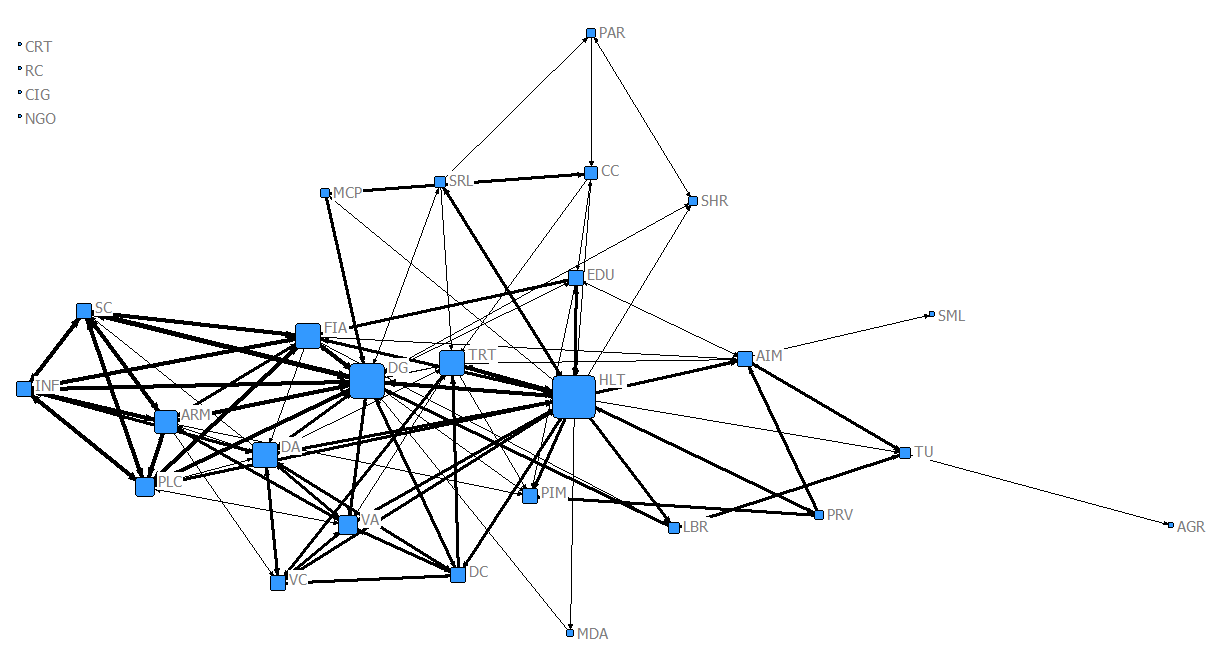 |
| Minab District  Isolate: NGO,RC,CIG,CRT | Roudan District  Isolate: NGO,RC, CIG,CRT |

| 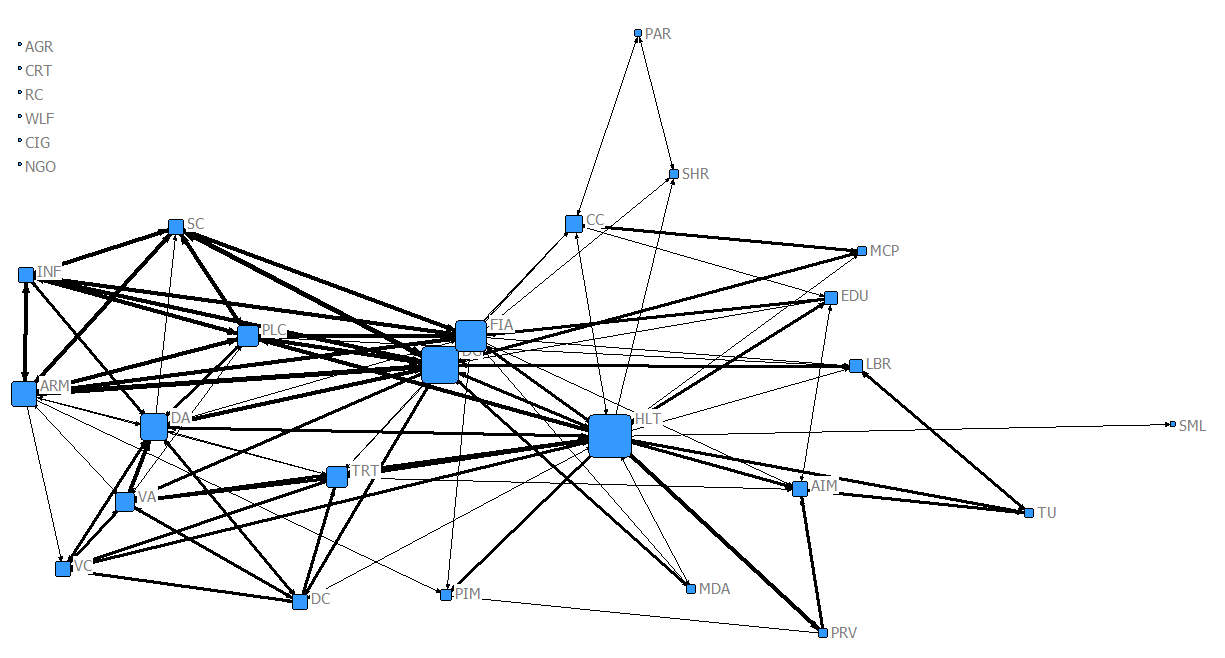 | 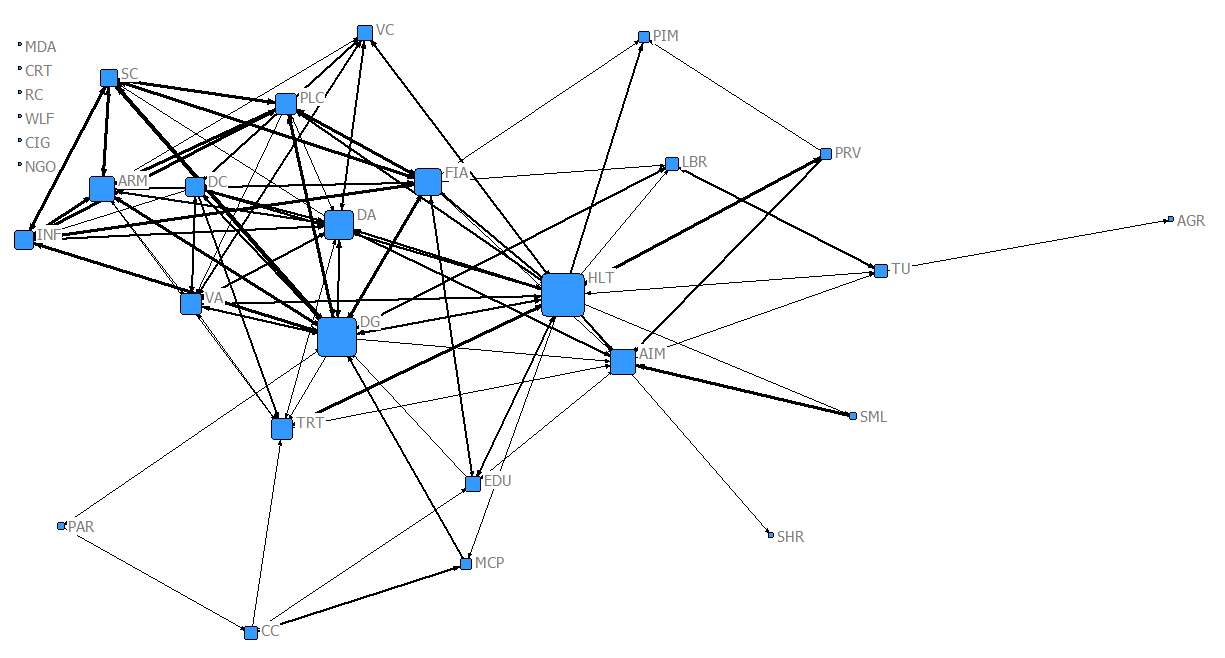 |
| --- | --- |
| Kahnouj District  Isolate: NGO,RC,CIG,CRT,AGR,WLF | Rudbar-e- Jonoob District  Isolate: NGO,RC,CIG,CRT,MDA,WLF |
| 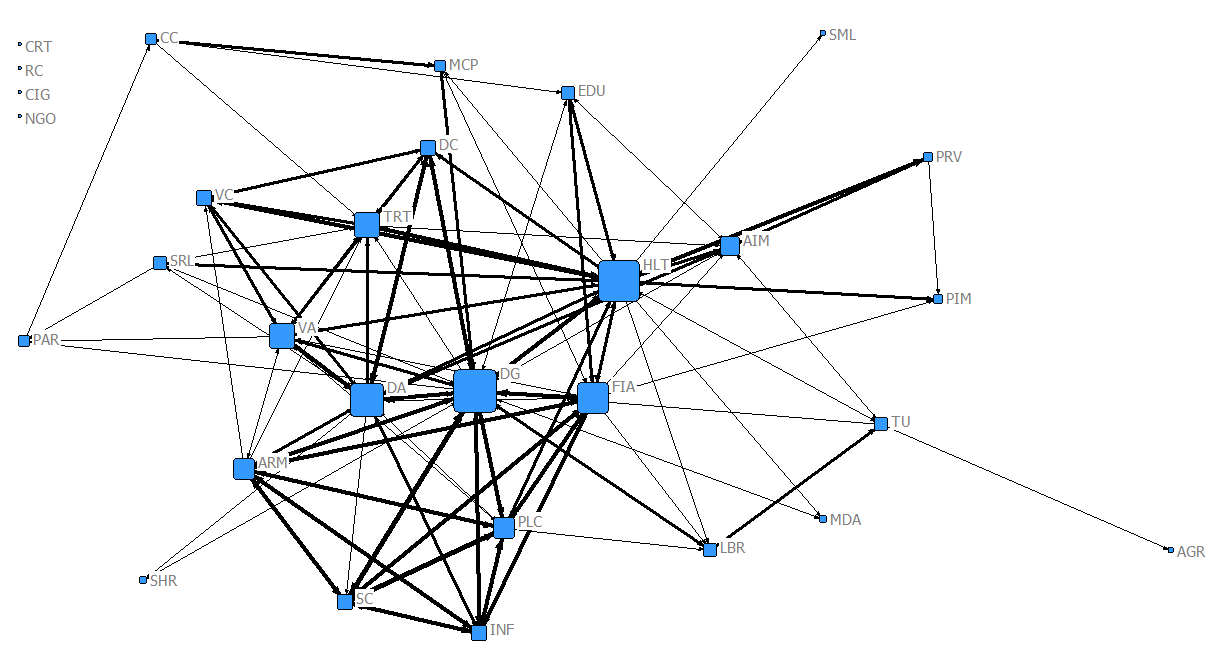 | 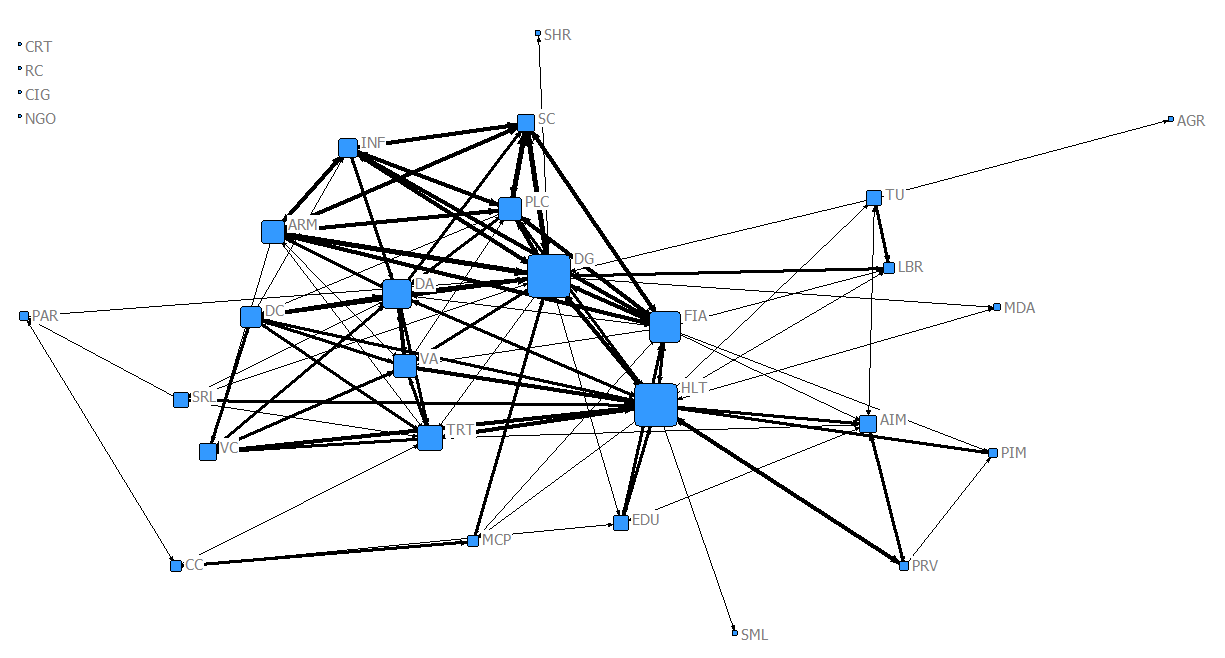 |
| Manoujan District  Isolate: NGO,RC,CIG,CRT | Qaleh Ganj District  Isolate: NGO,RC,CIG,CRT |

Additional file 2: Maps of stakeholders’ networks related to immigrants’ and refugees’ access to services and control their movement in the field of malaria elimination among endemic districts of Iran in 2016-2017
